# Aerodynamic Characteristics of the Ventilated Design for Flapping Wing Micro Air Vehicle

**DOI:** 10.1155/2014/410749

**Published:** 2014-02-06

**Authors:** G. Q. Zhang, S. C. M. Yu

**Affiliations:** ^1^Aerospace Engineering Division, School of Mechanical and Aerospace Engineering, Nanyang Technological University, Singapore 639798; ^2^Academic Division, Singapore Institute of Technology, Singapore 179104

## Abstract

Inspired by superior flight performance of natural flight masters like birds and insects and based on the ventilating flaps that can be opened and closed by the changing air pressure around the wing, a new flapping wing type has been proposed. It is known that the net lift force generated by a solid wing in a flapping cycle is nearly zero. However, for the case of the ventilated wing, results for the net lift force are positive which is due to the effect created by the “ventilation” in reducing negative lift force during the upstroke. The presence of moving flaps can serve as the variable in which, through careful control of the areas, a correlation with the decrease in negative lift can be generated. The corresponding aerodynamic characteristics have been investigated numerically by using different flapping frequencies and forward flight speeds.

## 1. Introduction

**Figure 1:**
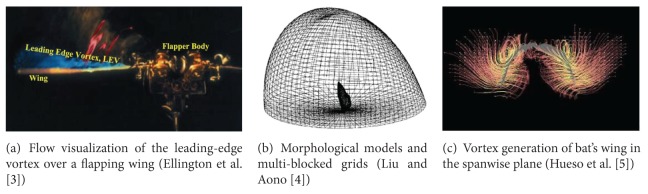
The presence of vortices in simulated airflow around the flapping wing model.

Micro air vehicles (MAVs) have the potential to revolutionize our sensing and information gathering capabilities in areas such as environmental monitoring and homeland security. In classical stationary wing theory, the tip vortices (TIVs) are seen as wasted energy; in flapping flight, they can interact with the LEV to enhance lift without increasing the power requirements [1, 2]. Just as referred by Ellington et al. [3] in Figure 1(a) (Nature 384: 626–630, 1996), to support the body weight, the wings typically produce 2-3 times more lift than can be accounted for by conventional aerodynamics. Liu and Aono [4] presented computational fluid dynamics (CFD) methods to study the insect hovering aerodynamics in Figure 1(b), which was performed using a biology-inspired dynamic flight simulator that integrated the modeling of realistic wing-body morphology, the modeling of flapping-wing and body kinematics, and an in-house Navier-Stokes solver. The corresponding results can not only give an integrated interpretation on the similarity and discrepancy of the near- and far-field vortex structures in insect hovering, but also demonstrate that the methods can be an effective tool in the MAVs design. Hueso et al. [5] provided the visualized results that emphasize the presence of vortices in simulated airflow around a motion-captured bat model in Figure 1(c). It shows a vortex in the spanwise plane (streamwise vorticity) captured over the wing in the flight of the bat.

Hu et al. [6] had conducted experiments to explore the potential applications of compact, gearless, and piezoelectric flapping wings with the wing size, stroke amplitude, and flapping frequency within the range of actual insect characteristics for the development of novel insect-sized, flapping-wing-based nanoair vehicles (NAVs). A digital particle image velocimetry (PIV) system was used to achieve phase-locked and time-averaged flow field measurements to quantify the formation and separation processes of the leading-edge vortex (LEV) structures on the upper and lower surfaces of the flapping wing in relation to the phase angle. It was found that the wake vortices in the cross plane at 50% wingspan would form concentrated vortex structures. As they travel downstream, the concentrated anti-clockwise and clockwise wake vortices were found to cross over at first and then align themselves in two rows with the clockwise (negative) vortices at above and anticlockwise (positive) vortices below, which is a typical von Karman vortex street.

Nagai et al. [7] also conducted the experimental and numerical studies to investigate the aerodynamic characteristics of a flapping wing of an insect in forward flight. Unsteady aerodynamic forces and flow patterns were measured using a dynamically scaled mechanical model in a water tunnel. The results indicated that these aerodynamic mechanisms had an effect on the aerodynamic characteristics of the flapping wing in forward flight; however, these mechanisms function differently during the up- and downstroke, for different stroke plane angles and for different advance ratios.

Singh and Chopra [8] used an experimental apparatus, with a biomimetic flapping mechanism to measure the thrust generated by a number of insect-based hover-capable flapping wings designs at different pitch settings. The wing mass was found to have a significant influence on the maximum frequency of the mechanism because of a high inertial power requirement. All the wings tested showed a decrease in thrust at high frequencies. In contrast, for a wing held at 90 deg pitch angle, flapping in a horizontal stroke plane with passive pitching caused by aerodynamic and inertial forces, the thrust was found to be larger. Sällström et al. [9] used the stereoscopic particle image velocimetry to investigate the airflow generated by two pairs of flapping Zimmerman planform wings under hovering conditions. The results indicate that the less stiff of the two wings sheds several vortices each half stroke, which may indicate that the wing stalls more rapidly in the beginning of each half stroke than the stiffer wing, in spite of a lower angle of attack. Mahardika et al. [10] conducted the experiments showing that the outer parts of the separated wings are able to deform, resulting in a smaller amount of drag production during the upstroke, while still producing relatively greater lift and thrust during the downstroke.

Phillips and Knowles [11] presented the experiment to investigate the effects of varying flapping kinematics on the mean lift produced by an insect-like flapping wing in hover. Results revealed that mean lift scaled with *f*(1.5) and varied proportionally with Theta. A pitch reversal advanced by up to 5 percent of the flapping period relative to stroke reversal was found to maximize mean lift, and delayed pitch reversals were detrimental to mean lift. Of the parameters tested, mean lift was also maximized for alpha(mid) = 45 degrees and Theta = 8.6 degrees. Lua et al. [12] were motivated by the works of Dickinson et al. [13] (Science 284: 1954–1960, 1999) and Sun and Tang [14] (J Exp Biol 205: 55–70, 2002) which provided two different perspectives on the influence of wing-wake interaction (or wake capture) on lift generation during flapping motion. They took a more fundamental approach to study the effect of wing-wake interaction on the aerodynamic force generation by carrying out simultaneous force and flow field measurements on a two-dimensional wing subjected to two different types of motion. These results suggest that wing-wake interaction does not always lead to lift enhancement, and it can also cause lift reduction. Mazaheri and Ebrahimi [15] had also investigated the aerodynamic performance of a flexible membrane flapping wing. Results indicated that the thrust increases with the flapping frequency. An increase in the wind tunnel speed and flow angle of attack leads to a reduction in the thrust value and increases the lift component.

To investigate aeroelastic effects of flexible wings (specifically, wing's twisting stiffness) on hovering and cruising aerodynamic performance, a flapping-wing system and an experimental setup were designed and built by Mazaheri and Ebrahimi [16]. Results show how elastic deformations caused by the interaction of inertial and aerodynamic forces with the flexible structure may affect specific power consumption. Based on unsteady numerical simulations, Elarbi and Qin [17] had studied the hovering capability of flapping two-dimensional tandem wing sections inspired by a real dragonfly wing configuration and kinematics. The results suggested that the longer time pitch rotation with the period of 80% of the overall flapping period is closer to the force calculations obtained of a balanced flight.

Using bird flight as the inspiration, the ventilated wing would apply the concept mentioned above and thus focus on “ventilating” the wings during the upstroke motion in hope of reducing any negative lift produced increasing net positive lift for each cycle. As the name suggests, a reduction in wing span would require a more complex mechanism to be built, which is meant to be kept as simple as possible.

## 2. Experimental Setup and Flapping MAV Design Concept

**Figure 2:**
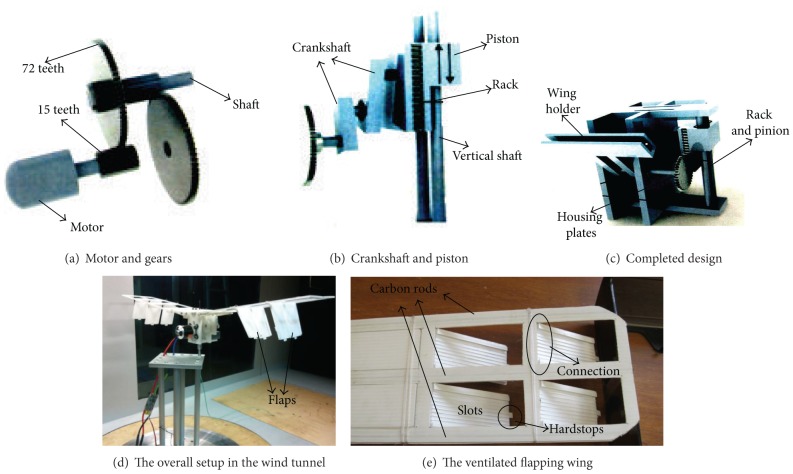
Experimental setup and ventilated flapping wing.

Based on the new flapping wing rotor design concept, a micro flapping wing rotor test model was designed and built as shown in Figure 2. The experimental procedure consisted of five primary components:

1. a high performance Tahmazo ER282610 brushless motor and a Lithium battery, to supply power for the model: this motor is a powerful 540 watt suitable for flying models up to 1.8 kg the speed is at 870 rpm/volt with the battery supplying a minimum of 1 V and up to a maximum of 11.1 V;
2. a gear mechanism, to decrease the rotation speed: two gears each of module 0.5 with the output/input gear are consisting of 75/15 teeth; the gear ratio is 4.8;
3. crankshaft, short off-centered bar linked to driving rod to transform the rotation to vertical linear motion as shown in Figure 1;
4. a rack and pinion actuator is used to convert the linear motion back to rotational motion;
5. wing holder is designed with a 3 mm slot in which the wings can be attached with screw holes included to firmly attach the wings into the wing holder if necessary.

In the creation of the ventilated wing, a series of slots have to be created on the wings. These slots should have the ability to be opened and closed either through active or passive control mechanisms during the flapping cycle. During the downstroke, the slots should ideally remain fully closed to represent the full wing area and generate maximum positive lift; as the upstroke begins, the slots should open out and allow “ventilation” to occur within the wings by reducing the exposed wing area, drag, and hence negative lift generated in the process. These slots areas would thus serve as the variable in which, through careful control of the areas, a correlation with the decrease in negative lift can be generated.

The major focus lies both in the design of the mechanism and the lift forces generated at the wings. Therefore, much attention should be paid to the wing design with the preliminary design for the wings shown in Figure 2(e). It is built from polypropylene corrugated sheets which is light in weight yet relatively tough. Additionally, each wing is fitted with three carbon rods, each running through the ends and middle. The carbon rods act as stiffeners to increase the rigidity of the wing and ensure as much as possible zero twist during the flapping maneuver. Thus, to not complicate any lift forces generated, it is essential for the wing to maintain a zero angle of attack with zero twist for both the upward and downward motions. The wing is initially made with a half wing span of 266 mm, areas of 0.0273 m^2^, aspect ratio (AR) of 2.58 with a rectangular shaped throughout and outer edge shaped neatly, and flapping frequency *f*, 2 to 4.96 Hz.

## 3. Simulation Setup

### 3.1. The Ventilated Wing Design

**Figure 3:**
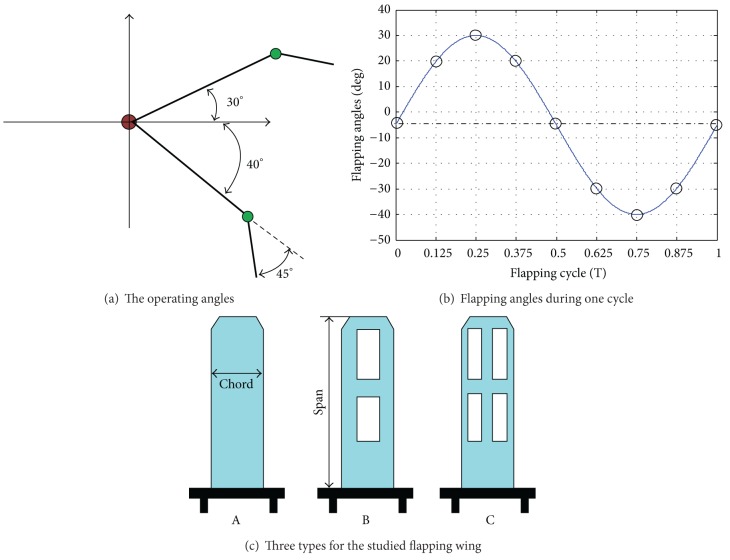
The operating process and wing shape type for the flapping mechanism.

Figures 3(a) and 3(b) show the operating angle of the flapping wing during one flapping cycle.  Figure 3(c) shows the three typical wing models, which were used in simulation.

### 3.2. CFD Method and Boundary Condition

**Figure 4:**
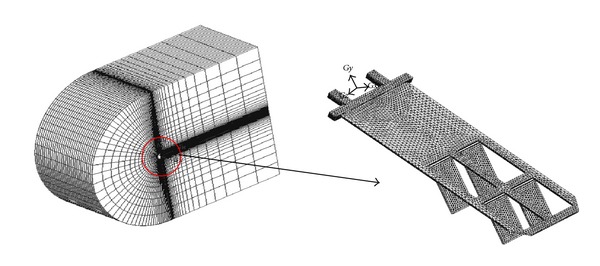
Decomposition of the computational domain.

The simulation is conducted using the ANSYS/FLUENT V.6.3.26. When modeling the whole aircraft, the results will be significantly affected by the quality of the mesh. As shown in Figure 4, the number of grid points is 120 × 56 × 60 in the tangential, radial, and spanwise directions, respectively. A nondimensional parameter that is widely used to describe the wing kinematics of flying birds and insects is the Strouhal number, Str = *fA*/*U*_*∞*_, which divides flapping frequency (*f*) and stroke amplitude (*A*) by the forward flying speed *U*_*∞*_.

For the present study, as shown in Table 1, the incoming flow velocity or forward flight speed is *U*_*∞*_ = 1.4  m/s; the chord length of the piezoelectric flapping wing is *c* = 103 mm. The flapping frequency of the piezoelectric flapping wing is *f* = 2  Hz~4.96 Hz. The peak-to-peak amplitude of the piezoelectric flapping wing at middle wingspan was found to be *A* = 84.05 mm. Following the work of Triantafyllou et al. [18] and Taylor et al. [19] to use the peak-to-peak flapping amplitude at middle wingspan to calculate the equivalent Strouhal number (Str) based on *f* = 4.96  Hz was found to be 0.298, that is, Str = 0.298, which is within the optimal range of 0.2 < Str < 0.4 usually used by flying birds and insects and swimming fishes.

In the numerical simulation, the relative pressure is adopted instead of absolute pressure. By using velocity inlet and pressure outlet boundary conditions as well as density-based explicit solver, simple iteration is adopted with large Eddy simulation (LES) turbulence model and bounded central differencing solution controls.

## 4. Results and Discussion

### 4.1. Comparison of the Simulation and Experimental Results

**Figure 5:**
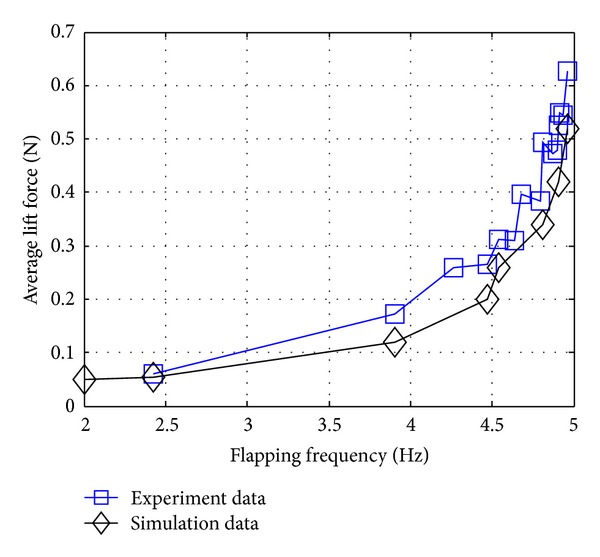
Average lift force based on the different flapping frequencies comparing with the experiment.

Figure 5 shows the average lift force produced by the parallel flapping wing (C-type model) as functions of the different flapping frequency (*f* = 2~4.96 Hz) without airflow introduction. In the results shown in Figure 7, it revealed that the experimental and simulation results can be seen to match in terms of trend. While they both display quadratic patterns, the experimental results can be seen to display more tortuous trend at higher frequencies (*f* > 4.3 Hz). In terms of magnitude, experimental results always show little bigger than the simulation. It can be attributed to the presence of four hardstops installed on the moving slots. In order to avoid creating negative volumes during flapping simulation, the corresponding hardstops had been removed. And the ground effect for the experiments should also be taken into consideration. Finally, the simulation has set the flapping wing to be entirely rigid body, ignoring the flexibility effects.

In addition, it can also be found that, in all the studied flapping frequencies, the average lift force produced by the single flapping motion will increase with the increasing flapping frequency monotonically.

**Figure 6:**
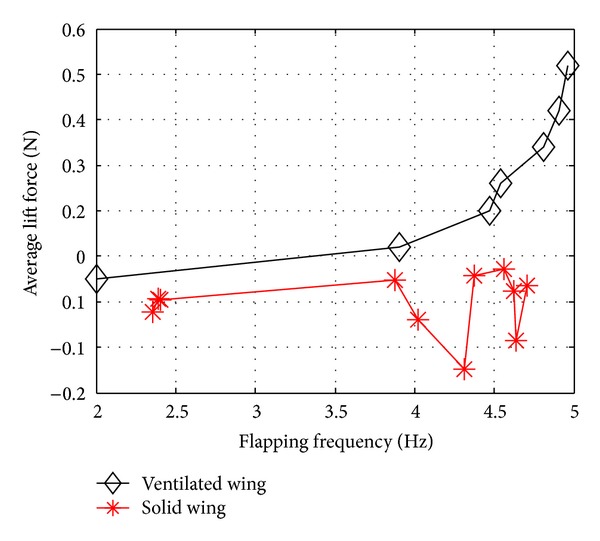
The average lift force characteristic for the ventilated wing versus solid wing.

As shown in Figure 6, the results for net lift force generated for the solid wing can be seen to fall very close to zero with all results falling within a band ±0.15 N from zero line. There is also no clear trend of net lift generated increasing with increased frequency with negative net lift still being generated at higher frequencies.

However, at the first glance for the case of a ventilated wing, as expected, it can be clearly seen that all the results for net lift force are positive which immediately justifies the effect created by the “ventilation” in reducing negative lift force from the upward stroke. A clear trend is also present whereby net lift force increases with increasing frequency. This increase in net lift can be seen to be linear from 2 to 3.8 Hz after which it starts increasing exponentially with frequency. At the highest frequency tested (4.96 Hz), as high as 0.63 N is generated with the plot predicting even steeper increases in net lift for frequencies above 4.96 Hz.

Table 2 shows the enhancement in net lift using ventilated wing compared to solid wing at selected frequencies. Besides depicting the exponential rise in lift force as frequency increases, the percentage increase shown in the table also serves to provide an initial indication of the frequency in which the ventilated wing mechanism starts activating fully which can be observed at about after 4 Hz (increase by 107.34%).

**Figure 7:**
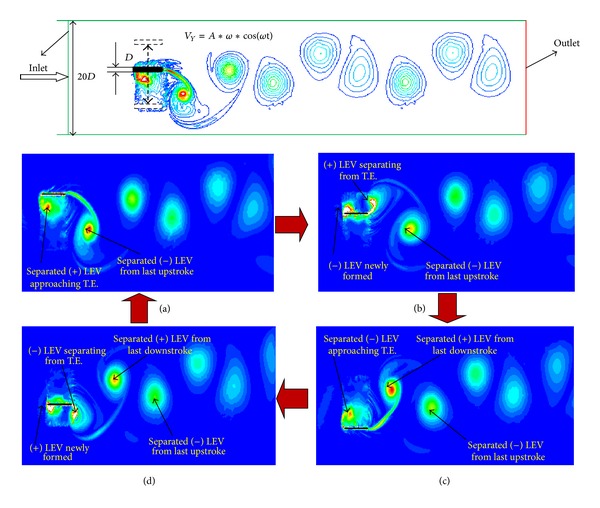
2D vortex shedding on the chordwise direction at the different positions. (a) Wing at the upmost position: downstroke starts. (b) Wing at the neutral position, during downstroke. (c) Wing at the bottom most position: upstroke starts. (d) Wing at the neutral position, during upstroke.

In order to investigate the mechanism of 3D flapping wing, firstly, we conducted the 2D flapping wing model. Figure 7 shows the vortex shedding at four typical positions: (a) upmost position (i.e., at the end of upstrokes or beginning of downstrokes); (b) neutral position during downstrokes; (c) bottom most position (i.e., at the end of downstrokes or the beginning of upstrokes); (d) neutral position during upstrokes, respectively.

As shown clearly in Figure 7(a), when the flapping wing was at its upmost position to start a downstroke, the anticlockwise (positive) LEV newly formed on the lower surface of flapping wing by the previous upstroke was found to be intensified rapidly in strength and separated eventually. It would move downstream approaching the trailing edge of the wing. And we also can observe that the separated clockwise (negative) LEV was separated and hung to the trailing edge of the flapping wing. The smaller and weaker wake vortex structures were found to be dissipated rapidly and eventually vanished at further downstream, identical to the typical Karman vortex street configuration. After the upmost position, the flapping wing reached the neutral position during the downstroke. From Figure 7(b), we can see that, during the downstroke, the clockwise LEV was gradually formed on the upper surface near the leading edge of the wing. At mean time, the anticlockwise LEV on the lower surface began to be separated, and it will be pushed to move further downstream gradually. Meanwhile, the newly formed clockwise LEV was found to be strengthened itself and stayed attached to the leading edge of the flapping wing. And the corresponding separated clockwise LEV formed by the previous upstroke has totally broken away from any edges of flapping wing and stepped into the downstream. As revealed in Figure 7(c), the newly formed clockwise LEV developed its structure and strength; it has covered almost 90% upper surface of the wing at the bottommost position, and it was found to be detached from the upper surface and approaching the trailing edge of the wing. Meanwhile, the separated anticlockwise (positive) LEV created by the previous downstroke was separated and hung to the trailing edge of the flapping wing. At the end of the upstroke of the flapping circle, shown in Figure 7(d), a new anticlockwise (positive) LEV has been formed on the lower surface of the flapping wing, and the separated clockwise LEV on the upper surface would shed from the trailing edge of the wing. Other shed vortexes will become smaller and weaker and be vanished eventually. This process (downstroke and upstroke) would repeat in cycles. Finally, the clockwise and anticlockwise vortexes were shed alternatively in the wake of the flapping wing.

**Figure 8:**
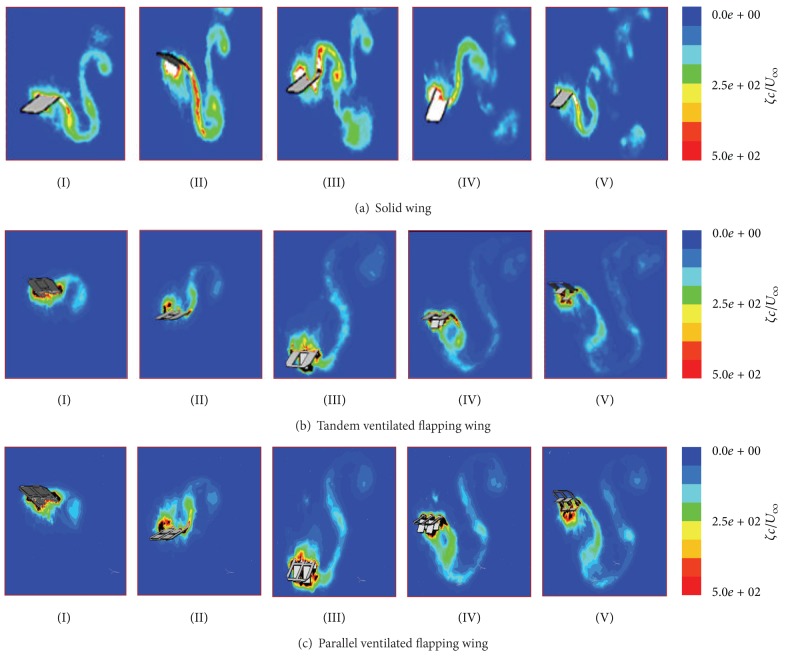
Contours of normalized vorticity magnitude *ζc*/*U* at different time and at zero angle of attack (AOA).

In Figures 8(a), 8(b), and 8(c), the LEV vortex forming and shedding for the solding wing and two typical ventilated wings have been shown. Although they have the same general trend, including the forming and shedding, due to the presence of slots, it can make the flow pattern become more complex. From Figure 8(a), we can see the LEV vortex has formed earlier and easier than the other two ventilated wing cases, and the shed vortex shows more islated with each other. Different from the solid wing case, the LEV vortex formation and shedding for the ventilated wing seem to need more time. Except this, the period is also relatively longer than the solid wing, and the upmost and downmost impacted region extended five times as long as the flapping amplitude A. The shed vortexes are also connected mutually with the long “tails.”

**Figure 9:**
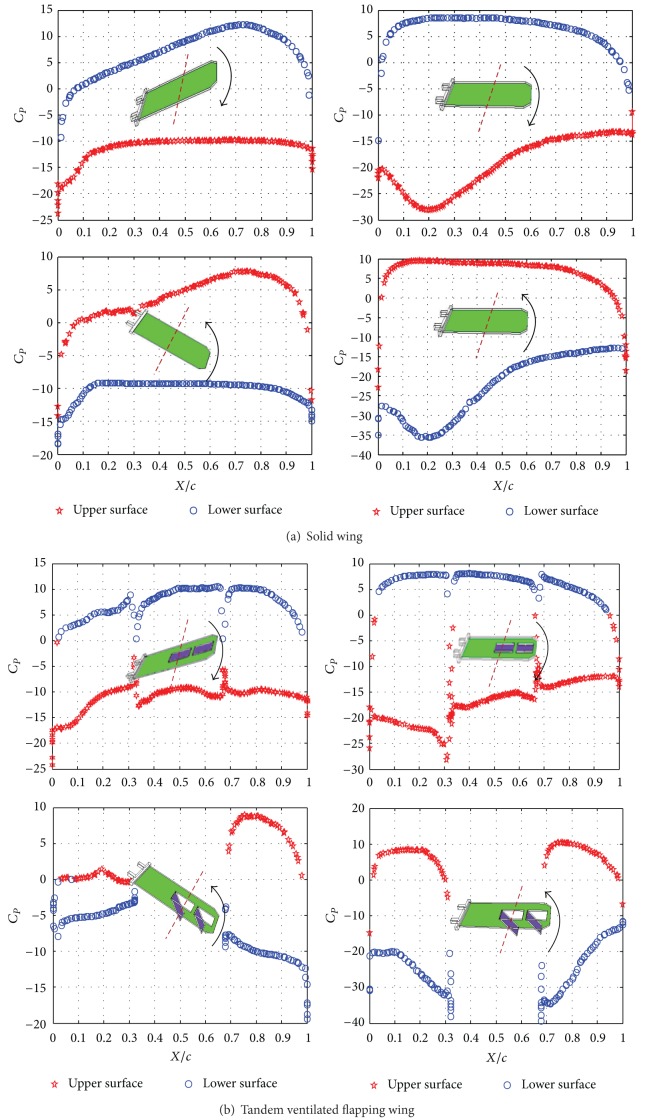

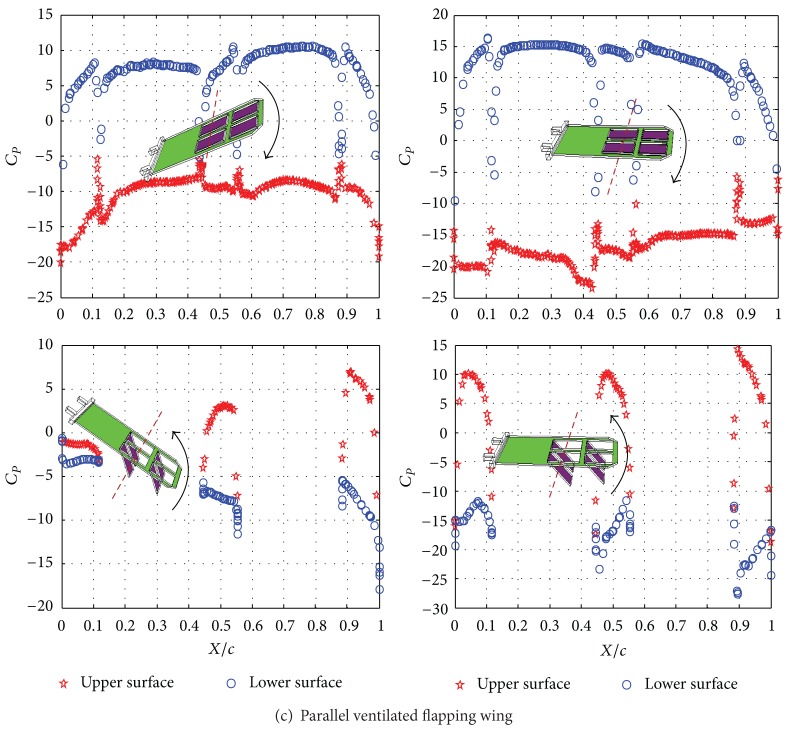
Pressure coefficient (*C*_*P*_) at *t* = 0.25 T, 0.5 T, 0.75 T, and T across the 50% chord length.

The pressure coefficient is negative for pressure less than the free stream, which may occur on the top or bottom surface of the wing or canard. The data from the simulation is expressed with respect to the local chord length *c*.  Figure 9 shows the pressure coefficient (*C*_*P*_) distribution on the solid wing (a), tandem ventilated wing (b), and parallel ventilated wing (c) at *t* = 0.25 T, 0.5 T, 0.75 T, and T, respectively, based on the four typical flapping positions.

Comparing with Figures 9(a), 9(b), and 9(c), for the solid wing case, due to the fact that it did not include any slots, so the corresponding *C*_*P*_curves are showing much smoother than the other two ventilated flapping wings. And the area of pressure curves of solid wing is entire and blank. Comparing with the solid wing, the area of *C*_*P*_ curves of tandem ventilated wing has been divided into three parts: main wing and two slots (seen in Figure 9(b)). And due to the presence of slots, the area of *C*_*P*_ curves of parallel ventilated wings has also been divided into five parts: main wing and four slots (seen in Figure 9(c)).

When the wing starts to flap up, the upper surface of the wing is actually playing the role of “the lower surface.” Contrary to the conventional theory, the higher pressure region is on the top surface of the wing (upper surface), and the lower pressure region is on the bottom of the wing (lower surface). So the upstrokes always generate the negative lift force for the flapping wing. However, after the upstroke starts, both the upper and lower surfaces will become reversed. So the downstrokes always generate the positive lift force. Consequently, there is, a common characteristic, that is *C*_*P*_ curves of the lower surface are always on the top of the upper surface during the downstrokes, and *C*_*P*_ curves of the lower surface are always on the bottom of the upper surface during the upstrokes.

However, we should not ignore the moving slots, because in all the above figures, we only focus on the entire movement, not specially considering the special time of slots movements. For it will open and close during the very beginning upstroke and downstroke, there will exist a short time that the *C*_*p*_ curves of the upper and lower slots surface have a contrary trend with the other common flapping time. When the main wing begins to flap up and down, the slots will also begin to open and close (moving down and up) immediately. In this short time (*t* = 0.04 T), the pressure on the upper slots surfaces actually should be higher and lower than the lower slots surfaces, respectively. Only after the slots had completed their own motion and began to flap together with the main wing, the *C*_*p*_ distribution would be changed into the above figures trends.

**Figure 10:**
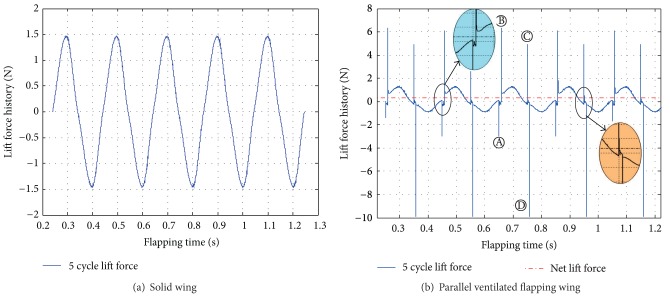
Lift force history during five flapping cycles.

Figure 10(a) shows the lift force history during five flapping cycles. The static angle of attack (AOA) of the flapping wing was set to be zero (i.e., AOA = 0 deg). Due to the downstroke and upstroke motion is repeated. The uniform velocity of the incoming flow was steady, so the maximum was similar to the minimum in values. As a result, the curves have been shown symmetrically, and the corresponding net lift force always fluctuated near the zero value. And compared with the other two typical ventilated flapping wings, the lift curve is much smoother and more regular (will be discussed as follows).

As shown in Figure 10(b), the lift force history for the parallel ventilated wing case had displayed different from the solid wing case; there are some rhythmic vibrations on the curve. The typical four peak values have been found and marked A, B, C, and D, respectively. Actually, these marks have further described the open and close motion with respect to the moving slots. Firstly, when the ventilated flapping wing begin to flap down, the slots rooted on the main wing would not only have to flap down with the main wing, but also begin to close (moving up); although the downstrokes should create the positive lift force, due to the fact that the small slots have accounted for almost 85% of the wing area, the small slots are actually the main lift parts for this kind of ventilated flapping wing. So when the main wing begins to flap down, the slots will begin to close; immediately, the total lift force will be jumped into the negative values, so the peak A was formed at this short moment. After this immediate start, the slots will be moved up slowly (*t* = 320 time step per time step = 2.5 × 10^−5^ s); when it completed the close motion, the upmotion has suddenly stopped. The negative force will also immediately jump into the positive, so this process has created the mark B. After half of the flapping cycle, the ventilated wing will begin to flap up. However, the fully closed slots will begin to open again at this time. Due to this motion, the negative lift force created by the upstroke has to jump into the positive, so this instant has created the mark C. After the open motion is completed (*t* = 320 time step), the fully opened slot will flap up together with the main wing, so the positive lift force has to jump immediately into the negative, creating the mark D at this moment. The downstrokes and upstrokes process (including the open and close motion of the slots) will repeat in cycles continuously. Finally, four typical marks will alternatively appear, respectively, in the whole flapping cycles.

## 5. Conclusion

Using bird flight as the inspiration, three types of the flapping wing have been investigated by using the dynamic mesh method. The corresponding conclusions have been drawn as follows.

The results for net lift force generated for the solid wing can be seen to fall very close to zero with all results falling within a band ±0.15N from zero line. There is also no clear trend of net lift generated increasing with increased frequency with negative net lift still being generated at higher frequencies. While for the case of a ventilated wing, all results for net lift force are positive which immediately justifies the effect created by the “ventilation” in reducing negative lift force from the upward stroke. A clear trend is also present whereby net lift force increases with increasing frequency.

For the LEV vortex forming and shedding for the solding wing and two typical ventilated wings, although they have the same general trend, including the forming and shedding, due to the presence of slots, it can make the flow pattern become more complex.

For the solid wing case, due to the fact that it did not include any slots, so the corresponding *C*_*P*_  curves are showing much smoother than the other two ventilated flapping wings. And the area of pressure curves of solid wing is entire and blank. However, the area of *C*_*P*_ curves of tandem ventilated wings will be divided into three parts: main wing and two slots. The area of *C*_*P*_ curves of parallel ventilated wing will also be divided into five parts: main wing and four slots.

For the solid wing, due to the downstroke and upstroke, motion is repeated. The uniform velocity of the incoming flow was steady, so the maximum is similar to the minimum in values. As a result, the curves have been shown symmetrically, and the corresponding net lift force always fluctuated near the zero value. While the parallel ventilated wing case had displayed different from the solid wing case, the lift curve will not be so smooth and regular; there are some rhythmic vibrations on the curve.

## Figures and Tables

**Table 1:** Specification of flapping wing model.

Case number	Flapping frequency *f* (Hz)	Free stream velocity (m/s)	Wing type
1	4.96	1.4	A
2	2~4.96	0	A
3	4.96	1.4	A
4	4.96	1.4	B
5	2~4.96	0	C
6	4.96	1.4	C

**Table 2:** Enhancement in net lift using ventilated wing compared to solid wing.

Frequency (Hz)	Increase in net lift (N)	Percentage increase (%)
2.5	0.059	—
3	0.0828	40.34
3.5	0.1063	28.38
4	0.2204	107.34
4.7	0.3721	68.83
